# Perception Accuracy of a Multi-Channel Tactile Feedback System for Assistive Technology

**DOI:** 10.3390/s22228962

**Published:** 2022-11-19

**Authors:** György Wersényi

**Affiliations:** Department of Telecommunications, Széchenyi István University, H-9026 Gyor, Hungary; wersenyi@sze.hu

**Keywords:** haptics, tactile feedback, vibrating transducers, perception, assistive technology, 07.10.Fq, 43.66.Wv, 87.85.Ox

## Abstract

Assistive technology uses multi-modal feedback devices, focusing on the visual, auditory, and haptic modalities. Tactile devices provide additional information via touch sense. Perception accuracy of vibrations depends on the spectral and temporal attributes of the signal, as well as on the body parts they are attached to. The widespread use of AR/VR devices, wearables, and gaming interfaces requires information about the usability of feedback devices. This paper presents results of an experiment using an 8-channel tactile feedback system with vibrators placed on the wrists, arms, ankles, and forehead. Different vibration patterns were designed and presented using sinusoidal frequency bursts on 2, 4, and 8 channels. In total, 27 subjects reported their sensation formally and informally on questionnaires. Results indicate that 2 and 4 channels could be used simultaneously with high accuracy, and the transducers’ optimal placement (best sensitivity) is on the wrists, followed by the ankles. Arm and head positions were inferior and generally inadequate for signal presentation. For optimal performance, signal length should exceed 500 ms. Furthermore, the amplitude level and temporal pattern of the presented signals have to be used for carrying information rather than the frequency of the vibration.

## 1. Introduction

Experiments targeting the exploration of limitations of sensation and perception performance of humans have been in focus for many decades. Sensation occurs when sensory receptors detect sensory stimuli. Perception involves the organization, interpretation, and conscious experience of those sensations. The most important modalities are visual and auditory. Furthermore, the haptic/tactile modality has recently gained importance. Augmented, virtual, and mixed realities; simulators; gaming scenarios; and various other assistive technology applications usually implement vibration as a feedback to extend or replace visual and auditory information. Introducing multi-modal feedback systems also increases the cognitive load of the users. Previously isolated research areas in electrical engineering, computer science, infocommunications, and psychophysical sciences are now merging under terms, such as cognitive infocommunications, tactile internet, metaverse, assistive technology, and similar concepts [1,2,3,4,5,6,7,8,9]. Recent developments include not only 3D audio and video playback, but also tactile input and output for a fully immersive user experience.

### 1.1. Tactile Modality

Vision is seen as the most important modality, followed by hearing, and, in interactions with the environment, tactile (touch) modality is third. The brain seldom processes environmental information sequentially using successive sensory modalities rather it simultaneously processes stimuli from several sensory modalities where sensory overload can occur [10,11]. Haptic sensing relates to any sense of touch and is a combination of two sensing abilities: tactile sensing (detection of force/vibrations on the skin, identification of a physical object’s features) and kinesthetics (detection of an object’s features via movement and muscle strength). Measures of tactile sensitivity for the human body, capabilities, and limitations of the tactile modality, along with applications of human tactile interfaces were already summarized in these papers.

Based on the response characteristics of the four mechanoreceptors in humans, we can determine the sensitivity in correlation with frequency. So-called FA I fibers respond to low frequency vibrations from about 5 Hz to 50 Hz (localized sensation of wobble or flutter sensation), whilst FA II fibers respond from about 50 to 700 Hz (buzz sensation). The latter is more important for applications using small-size vibrators (such as those used in cellphones) operating around some hundreds of Hz [12,13,14,15,16]. Hairless body parts are often tested, as humans have relatively little hair on the body in contrast to other mammals. However, the forearm can be one of the important body parts that can be covered with hair and still used for vibration perception (vibrating armbands, wristbands, smartwatches, etc.). The face, lips, trunk, hands, and fingers are generally more sensitive than the thigh or upper arm, calf, and foot. However, only a few of these can be used in applications for feedback: hands and fingers can be used for the most sensitive applications, and other extremities with decreased sensitivity. Tactile vibration is a change in pressure over time. Absolute vibration threshold experiments on the fingertips revealed that vibrations below 5 Hz and up to 5400 Hz can be detected. People are not equally sensitive to vibrations at different frequencies. Temporal distance means we can detect whether two tactile pulses appear to be simultaneous or successive in time. Temporal differences as low as 5 ms can be perceived. Touch is better than vision (25 ms) but worse than aural (0.01 ms). The minimum distance between two points on the skin also has a threshold. The minimal separation between two points is the best at the fingers, face, and palm. Lower and upper arms, however, are not optimal. Tactile sensitivity also declines with age (−1% per year in acuity levels). Interestingly, tactile spatial acuity and sensitivity are related to our genes and it is also correlated with hearing acuity.

### 1.2. Vibration Perception Thresholds

Haptic perception is the perceptual processing of inputs from multiple sensory systems of skin, muscles, and joints and it is active during the exploratory procedure of the environment, including artificially generated signals if present. Interaction between touch and other modalities can influence results, especially in the case of vision: they are intrinsically complementary (geometry and material).

Vibration perception threshold (VPT) is the threshold at which humans can perceive vibration. It is also widely used as a measure of nerve fiber function. It depends on vibrating frequency, amplitude, and the body part where transducers are attached [17]. Healthy volunteers have already been studied in the late 1980s and early 1990s regarding the influence of age, height, sex, and even smoking on the VPT values. Measurements were made on great toes, thumbs, and ankles. Height was a significant factor for toes and ankles but not for thumbs. Sex had no overall effect on toe or thumbs but there were differences regarding ankles [18]. VPT of fingers measured on forest workers was dependent on prior vibration exposure, and the most sensitive VPT was found between 63 and 125 Hz [19]. In another experiment, 202 healthy adults were tested at 100 Hz at six different measurement sites [20]. The effect of temperature on VPT values was also evaluated: variation in VPTs caused by temperature was only slightly greater than intraindividual variation from day to day. In this experiment, VPT was found to increase exponentially with age, more for men than women. Acceleration magnitudes at different body segments for different frequencies were measured on 65 young adults at 12 different frequencies and two intensities (3 and 5 mm amplitudes) on the head, hip, and lower leg [21]. Intensive magnitude perception was observed around 21 and 25 Hz for the limbs, around 11–25 Hz for the trunk, and light perception at 13–25 Hz on the head. At 5 mm amplitude, subjects reported discomfort in the foot region. These frequencies are way too low for small-size buzzers. Nowadays, integrated tactile-thermo vibrators are also available [22,23]. Recently, 924 adults’ VPT was measured on fingers and feet [24]. Increasing skin temperature affected VPTs in finger pulps, but not on feet. Height was only found to affect the VPTs of the foot (0.42 dB per cm at 250 Hz). Mean VPT values in decibels are relative to 10^−6^ m/s^2^ and are in the 105–120 dB region at 250 Hz for young adults where sensitivity is maximum. Sex, weight, and handedness did not affect the VPTs. These results indicate high variations of VPT values without a clear conclusion on the different body parts.

In addition to experimenting with different body parts, the whole body can also be used as a test object. Whole-body vibration thresholds were determined in three dimensions using sinusoidal vibrations for sitting and standing subjects (from 2 to 100 Hz) [25]. For vertical vibration of seated subjects, no significant differences were found between the responses of male and female subjects, but differences were found between perception thresholds for sitting and standing postures. The median threshold was approximately 0.01 m/s^2^ rms between 2 and 100 Hz. Perception thresholds for the x-axis and y-axis vibration were not different in either sitting or standing subjects. Subjects tended to be more sensitive to vibration when lying than when sitting or standing. The frequency of vibration did not affect perception thresholds for vibration durations of more than about 0.25 s. For seated subjects, whole-body vertical sinusoidal vibrations were tested at two vibration magnitudes and at two frequencies (5 and 20 Hz) [26]. A vibration magnitude needed to be reduced by more than about 10% for the change to be detectable by human subjects. Whole-body vibration reproduction systems would also require individual transfer function measurements, and compensation methods, especially in the case of multi-modal feedback [27].

In addition to frequency and amplitude, temporal features (patterns) of vibrations play a significant role. Temporal characteristics of vibration of a mobile device were tested in an experiment [28]. Six vibration stimuli of different lengths were applied and users scored a subjective perception level on a five-point scale. The results suggested that the optimal duration of the control signal should be between 50 and 200 ms in this specific case. Longer durations were perceived as irritating.

### 1.3. Assistive Technology

Assistive technology (AT) includes products, equipment, and integrated software solutions that enable or enhance possibilities and safety of daily living (especially during study, work, and mobility) for persons with disabilities. Tasks in product and software development are very similar and overlapping to those present in enhancing services in communication or entertainment of healthy individuals. Regarding tactile information, blind people make use of such developments, especially during navigation as an extension or replacement of audio feedback signals [29,30,31,32,33]. Persons with visual impairment generally use a cane to explore their surroundings and sense objects. Electronic aids and wearables may help them during navigation, but provided information is limited. In addition to the auditory modality, lots of haptic solutions were also introduced and tested. VibroVision, a vest projecting information about the area in front of the wearer using a two-dimensional tactile image rendered by an array of vibration motors, enables users to sense features, such as shape, position, and distance of objects in front of them [34]. The Sound of Vision project also tested multi-vibrator vests along with helmets, and headsets [35]. In addition to vests, special canes and camera-based headsets, belts were also developed (NavBelt [36,37], Kinect Cane [38], etc.).

Safety is the most important issue during navigation, thus, accessing auditory information from environmental sounds has to be maintained. Wearable travel aids, therefore, use some kind of tactile feedback as an extension of the auditory modality. On the other hand, for activities in a safe environment (i.e., working on a computer) more information could be delivered via multi-sensory and multi-channel feedback solutions [39,40]. Vibro-tactile displays in general convey messages by presenting vibrations to the user’s skin passively (i.e., Braille) or actively (i.e., vibrators). Guidelines are available for experiment setups and interface design based on neurophysiological and psychophysical data [41,42].

Although assistive technology usually refers to electronic aids for impaired people, it can also be used for entertainment (gaming), simulators, and military applications. Helmet-mounted tactile displays can be used by the military to alert soldiers to the direction and location of an event [43]. Here, accuracy on the head is important. Another important body part is the arm (hand and fingers). Fingertips are one of the most sensitive skin areas of the body, but their use is limited if the interface also uses fingers (keyboard or mouse); however, this can be a feedback point in other cases [44]. Vibrating armbands are commercially available and smartwatches also incorporate vibrators to alert users. The Myo armband is a one such example that has been used for tests [45,46,47,48]. An armband embedding four vibrating motors is enough to guide the wrist of an operator along a predefined path or to a target location of a robot [49,50]. The forearm and upper arm are good locations for vibrators. PneuHaptic is a pneumatically-actuated arm-worn haptic interface. The system triggers a range of tactile sensations on the arm by alternately pressurizing and depressurizing a series of custom-molded silicone chambers [51]. A wireless wearable 8-channel haptic armband was also tested for feedback [52]. Distributed patterns of mechanical waves around the limb were used. In this experiment, the following were measured: the sensory thresholds in eight locations around the forearm, user adaptation to sensation, user performance in discriminating multiple stimulation levels, and device performance using four spatial patterns. The sensation threshold depended on the actuator position and increased over time. The maximum resolution for stimuli discrimination was four. Using this resolution, four patterns with different spatial and magnitude properties were generated to evaluate the performance of subjects. The optimal vibration pattern varied among participants. Wearable stimulation devices (gloves, bracelets) can be used in rehabilitation engineering for therapy providing mechanical and vibratory input to stroke patients [53,54]. Imperceptible vibratory stimulation to the wrist can be used parallel with natural hand movements as well.

It is also common that assistive systems use multi-modal presentation of information, including visual, auditory, and haptic. The auditory perception is often coupled to tactile perception [55,56]. In an experiment, the influence of vertical whole-body vibrations on loudness perception was investigated. Frequencies 10, 20, 63, and 200 Hz were chosen to stimulate different tactile receptors. Three different vibration levels were used to study the influence of vibration amplitude. If an acoustic stimulus is accompanied by a vibratory stimulus, the level of acoustic stimulus is on average perceived one decibel higher. Surprisingly, this effect was independent of the vibration level.

Test results also showed that the addition of tactile feedback to a touchscreen significantly improves finger-based text entry, bringing it close to the performance of a real physical keyboard [57]. On the other hand, a non-touchscreen tactile wearable interface could be an alternative to touchscreens on wearable devices [58].

This paper presents a multi-channel tactile feedback system consisting of 8 vibrators placed on different body parts. Volunteers evaluated the perception accuracy of selected excitation signals in different experimental sessions. Section 2 introduces the hardware and software elements of the measurement system, including signal generation, vibration motor principles, and measurement procedure. Section 3 presents the results obtained in 2-, 4-, and 8-channel measurement sessions. In Section 4, results are discussed, highlighting the main findings and future perspectives. Finally, the last section concludes and summarizes the outcomes of the experiment.

## 2. Materials and Methods

The goal of this experiment was to test the properties and applicability of multi-channel tactile excitation signals on different body parts using a software-based digital audio workstation (DAW) to create and playback source files.

### 2.1. Measurement Setup

The measurement system’s hardware is based on a standard Win10 laptop computer connected to the Asus Xonar U7 external sound card via a USB hub. For signal generation, 96 kHz sampling frequency and 24 bit resolution were used on all channels. The output signal-to-noise ratio of 114 dB, flat frequency response up to 20 kHz, and the ASIO driver for low-latency communication maintained good quality analog signal reproduction for our purposes [59]. Accessing the analog signals is possible via a pair of RCA connectors (originally dedicated for the front left and right speakers) and via additional 3 stereo 3.5 mm jack connectors (dedicated for the remaining six audio channels). Instead of speakers, 8 micro button-type flat vibration motors were connected to the sound card’s connectors (see below). Although 6 channels are paired in stereo connectors, all channels can be controlled independently (Table 1). A control measurement was executed at 100 Hz, 500 Hz, 1 kHz, and 10 kHz using sinusoidal signals and measurement points at the connectors with maximum output power. Output levels of 0.93 V, 0.92 V, 0.90 V, and 0.89 V were measured, respectively, indicating almost equal output levels.

The software DAW “Reaper” was used for playback of 2–8 channels simultaneously according to the actual test session. It was selected based on pricing, easy installation, and effortless use of multi-channel playback (it also offers 60-days of trial usage with full functionality) [60]. The iZotope RX7 Audio Editor was used for signal generation. This program allows for the generation and manipulation of the sound from one or many audio tracks concurrently. In our case, monotic sound samples were generated and exported to wave files. These were then imported by the “Reaper” and were allocated to dedicated channels for independent playback.

### 2.2. Signal Generation

**Figure 1:**
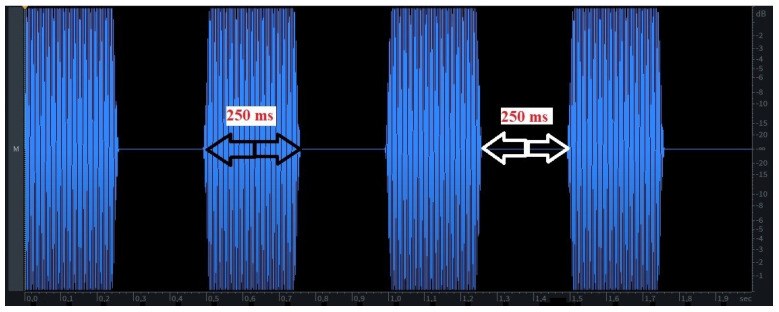
Example of a 2-s test signal pattern that has 250 ms of sinusoidal bursts of a given frequency followed by 250 ms of silence.

All signals (patterns) generated for the experiment are based on single frequency sinusoidal signals of different lengths (bursts), pauses (silence), and frequencies. All test signals have a length of 2 s and different patterns of sinusoidal bursts and pauses in between. Test signals can be played back in a loop to increase excitation time if needed. The length of bursts and pauses can be 100, 200, 250, 500, and 1000 ms. Figure 1 shows an example using 250 ms timing.

**Figure 2:**
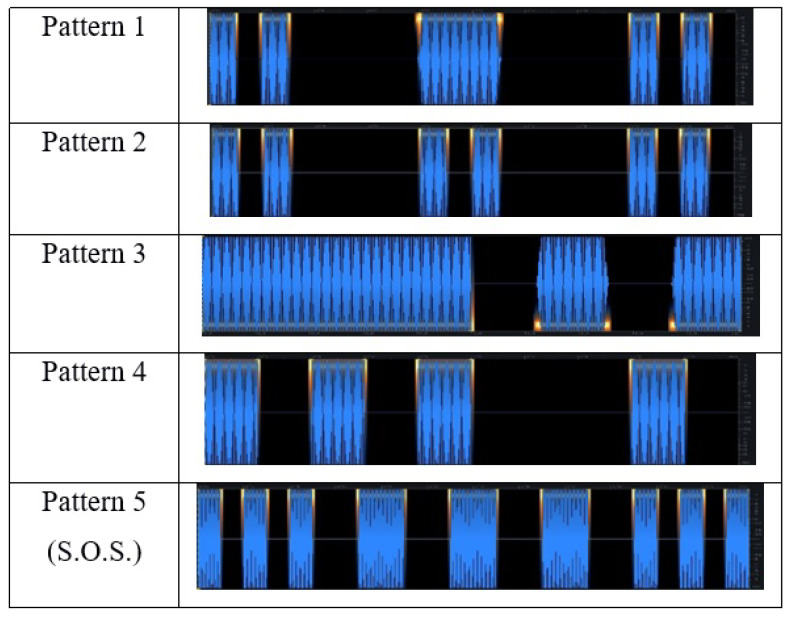
Patterns P1 to P5 used in the experiment for sessions 33–41.

If bursts and pauses have different lengths in the same signal, different patterns can be generated. Using the 235 Hz signal, five additional test signals with different patterns were created (Figure 2). The first uses two short 100 ms bursts, followed by a 300 ms, and two 100 ms. The second contains three pairs of 100 ms bursts. The third begins with a second-long sinus signal followed by two 250 ms bursts. The fourth contains four 200 ms bursts with 200 ms and 600 ms pauses. The final pattern is an S.O.S. signal composed of 100 ms and 200 ms bursts. An arbitrary number of patterns and signals can be generated and adjusted to a given experimental goal. In this experiment, test signals can be grouped as follows:

1. The 3 continuous pure sinusoidal signals (185 Hz, 235 Hz, 300 Hz);
2. The 4 intermittent 235 Hz sinusoidal signals (100 ms, 200 ms, 250 ms, 500 ms bursts and pauses, respectively);
3. The 5 different patterns (P1–P5).

### 2.3. Vibrators

Vibrating motors use moving masses to create vibrations. Small-sized “button-like” or “coin” vibrators have round-shaped masses moving along an axis. These types are very common, cost-effective, and accessible in a wide variety of sizes, usable frequencies, and sensitivities. The most important parameters are vibration strength (amplitude) and frequency range. Furthermore, we distinguish between DC-based and AC-based operations. In the case of DC motors, there is a nominal voltage level for operation that determines the nominal (optimal) frequency. Frequency is directly controlled by the voltage level. Linear Resonant Actuator (LRA) motors are AC motors, usually in a micro-size button format. DC motors require a DC/AC converter, thus we decided to use LRA motors for the experiment that can be directly connected to the output connectors of the sound card. LRA motors operate at maximal output level at the resonance frequency. This is the center frequency of transfer characteristics. The nominal operating frequency and impedance should match the output level and impedance of the sound card as much as possible. However, in the case of a mismatch, the operation can be still maintained with limitations in sensitivity. In the case of low-level signal reproduction, additional external audio amplifiers can be used.

**Figure 3:**
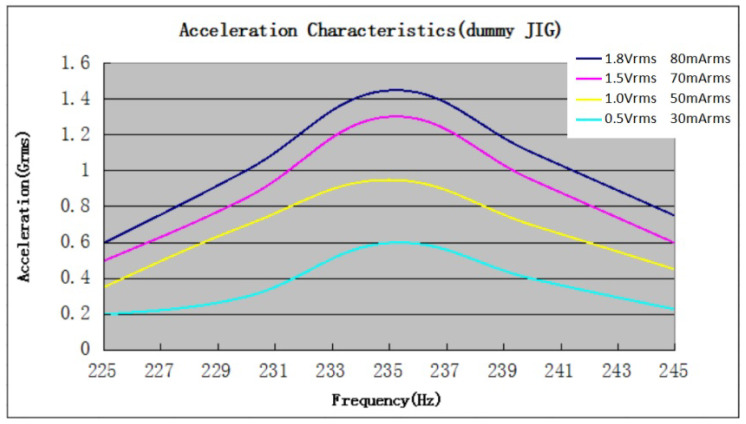
Linear vibrator frequency characteristics of the motors according to the manufacturer’s data sheet [61].

The sound card has a line output level of 1 V rms (2.83 Vpp) according to the data sheet. The output impedance of the sound card is between 32 and 150 Ohm, which usually matches the impedance of motors. After considering different available solutions, a set of Vybronics V-G0832013D type motors were purchased. It has an operating range from 0.1 V rms to 1.8 V rms (nominal) and an impedance of 22.5 Ohm [61]. It vibrates on the Z axis, which is perpendicular to the face of the vibration motor, making it ideal for battery powered mobile solutions and wearables. Based on the data sheet provided by the manufacturer, it has symmetric transfer characteristics with a center frequency of 235 Hz as shown in Figure 3. This frequency was selected as the basis for signal generation. When the output level of the computer and sound card were set to maximum, input voltage control measurements in the motor resulted in 0.68–0.71 V rms, which is below the optimum; however, vibrations were detectable at the extremities (see later).

### 2.4. Playback System

**Figure 4:**
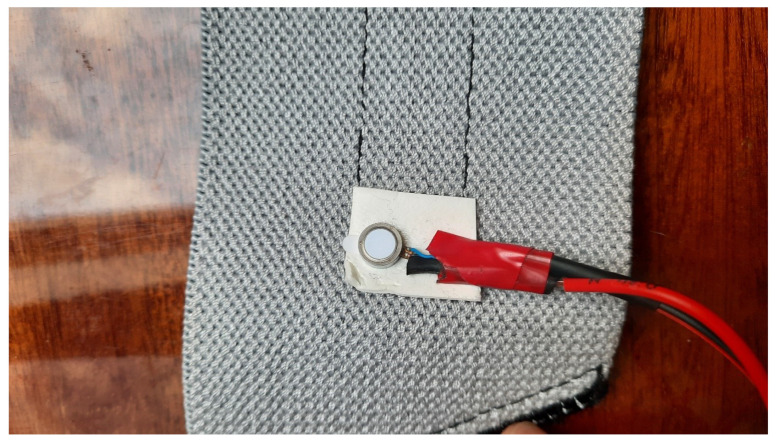
The vibrating motor fixed with double-sided tape.

**Figure 5:**
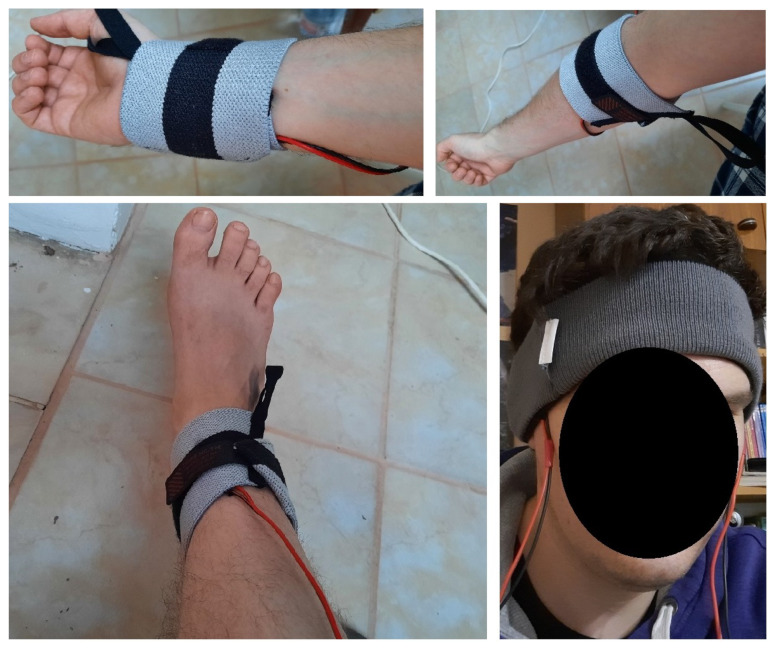
Fixing points on the human body.

The vibrators were fixed on commercially available sporty adjustable wristbands and a headband using double-sided tapes (Figure 4). The same type of wristband was used on the ankles, wrists, and arms. Figure 5 shows fixing points on the human body. Wrist refers to the point where watches or any regular wristband is worn. Ankle refers to a point right above the medial malleolus. Arms were excited on the inner side of the lower arm, near the elbow. The temporal fossa is a shallow depression on the side of the skull.

Based on the transfer characteristics of the vibrator, symmetrical damping in the amplitude of the vibration is predicted. In an empirical control measurement, a sinusoidal sweep signal was presented from 10 Hz to 1 kHz on the right wrist of 10 subjects. Frequencies below 150 Hz and above 400 Hz were undetectable. The frequency range between 200 and 270 Hz seemed optimal for the experiment. In the transition domain between 150–200 Hz and 270–400 Hz, low amplitude vibrations occurred that may or may not have been detected by the subjects due to increased damping. In a second preliminary experiment, both the left and right wrists were used for a comparative evaluation of sinusoidal signals of different frequencies. The goal of this was to determine two frequencies below and above 235 Hz that would be used in the main experiment. Output levels were set as identical on both channels by damping of 235 Hz signal in the software. Having the 235 Hz signal as the reference on the right wrist, the frequency of the left wrist signal was modified to the point where a difference in amplitude could be observed. Frequencies between 185 Hz and 300 Hz could not be distinguished from the 235 Hz signal. Results of these preliminary experiments were used to generate the final measurement signals. Then, 185 Hz (+4.85 dB) and 300 Hz (+5.00 dB) were selected for the main experiment as comparison signals to 235 Hz. This kind of equalization was only used for sessions 2 and 3 for the continuous signals. In all other sessions, the highest output levels were set on all transducers.

### 2.5. Measurement Procedure

**Figure 6:**
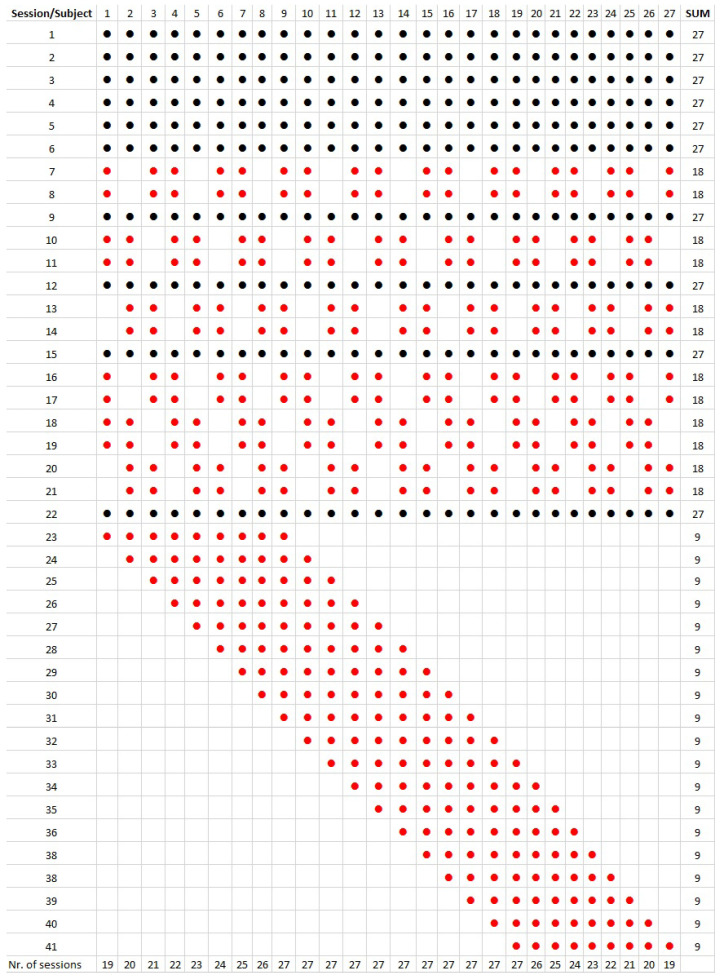
Graphical representation of the test sessions. Each column represents one subject, and each row corresponds to a session. Black dots refer to mandatory sessions evaluated by all 27 subjects. Red dots refer to non-mandatory sessions distributed among the subjects equally. Each 2-channel and 4-channel session was evaluated by 18 subjects, and each 8-channel session by 9 subjects. The last row shows the total number of sessions for each subject varying between 19 and 27.

In total, 27 subjects participated in the experiment (17 males, 10 females, mean age of 25.4 years). To reduce measurement time and demand, each subject had 19 to 27 sessions from the possible 41. 10 sessions were mandatory for every subject (No. 1–6, 9, 12, 15, and 22). The remaining sessions were selected to maintain equal distribution of the sessions. Figure 6 shows all sessions according to the procedure, including the total number of sessions for each subject. Since the main goal was to test the signals (sessions) rather than the subjects, evaluation is based on a comparison of the signals and body parts. Future work may include further subjects with reorganized sessions and an evaluation based on age, gender, handedness, etc.

**Figure 7:**
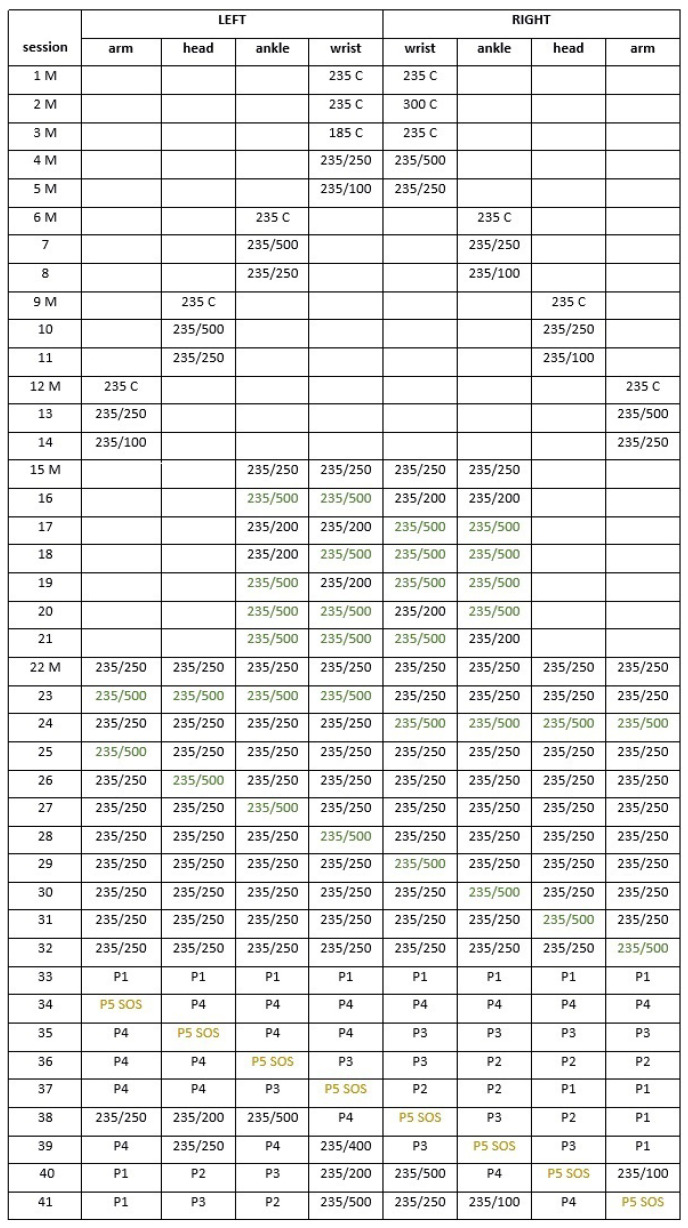
All sessions used in the experiment. M denotes mandatory sessions and C is for continuous signal. The first number in each cell corresponds to the frequency, followed by the length of the bursts and pauses in milliseconds. Colored characters help orientation in the table by highlighting the same signal. In sessions 33 to 41, P1 to P5 are the patterns based on Figure 2.

Figure 7 shows a list of all sessions used in the experiment. In the first column, M denotes mandatory sessions that were evaluated by all subjects. The first number in each cell represents the frequency, followed by the length of the bursts and pauses in milliseconds. C is for continuous signal. In rows 33–41, P1 to P5 are the patterns according to Figure 2. For example, session number 3 was mandatory, where the left wrist signal of a continuous 185 Hz was compared with a 235 Hz continuous signal on the right wrist. Session number 23 was optional, all 4 channels on the left side were 235 Hz bursts of 500 ms followed by 500 ms pauses; all 4 channels on the right side were the same 235 Hz bursts but of 250 ms length and pauses.

Although the number of channels could be 1–8, only 2-channel, 4-channel, and 8-channel signal presentations were included in the protocol according to Figure 7. Corresponding left and right transducers were active, however, they could have a different signal. Due to a very large number of possible presentation modes existing that have 12 signals and 8 channels, a restricted selection of 41 different sessions was included. This number is still relatively large in order for one subject to complete all of them, as it would result in a long experiment time per person and total. The goal of the experiment was to test the signals and fixing points, thus, subjects were allocated to different sessions to obtain an equal number of presentations. Using 8 channels, each session was evaluated by 9 subjects. Using 4 channels and 6 of the 2-channel sessions, evaluation was made by 18 subjects. Ten mandatory sessions were evaluated by all 27 subjects (see the rightmost column of Figure 6).

Subjects were seated in a silent laboratory environment. A short description of the task was given, followed by the placement of the transducers. After testing functionality, one experiment with one subject was divided into two halves: after completing half of the sessions a short break was inserted. Signal presentation was looped to a maximum length of 30 s composed of a 2-s element, but terminated after the subject delivered an answer. This happened usually between 8 and 20 s. Subjects responded orally to the experiment leader and responses were collected in a table manually. After the experiment, some informal questions were asked about general usability and experience.

## 3. Results

This section presents the results of evaluating subjects’ responses in 2-, 4-, and 8-channel sessions. Statistical results are presented in the Supplementary Materials.

### 3.1. Two-Channel Experiments

There are 14 2-channel sessions. No. 1–5 used the wrist as mandatory sessions. Furthermore, no. 6, 9, and 12 were also mandatory, using the 235 Hz continuous signal on the ankles, head, and arms, respectively. These 8 sessions have 27 responses each. Non-continuous signals in sessions 7–8, 10–11, and 13–14 were completed by 18 subjects.

#### 3.1.1. Wrists

The first test for every subject was always session no. 1 where both wrists were excited with the same 235 Hz continuous signal. All subjects could detect the vibration on both wrists and commented “no difference” between the two sides neither in frequency nor in amplitude, as expected (100%). The following two sessions used different frequencies and equalized amplitudes based on the preliminary tests. Using a 5 dB damped 235 Hz to compensate for the damping of the transducer, all subjects reported equal amplitudes of the signals. Detecting differences in frequency was more difficult: exactly two-thirds of the subjects (18) could not detect any difference neither between 185 Hz and 300 Hz (session 2) nor between 185 Hz and 235 Hz (session 3), while the remaining 9 subjects could sense a difference in frequency. Sessions 4 and 5 tested temporal attributes of the burst signals. Then, 500 ms “longer” bursts and 100 ms “shorter” bursts were compared with 250 ms bursts. Longer bursts were easier to detect, as 22 out of 27 subjects felt a difference, but the result was only 13 out of 27 in the case of shorter bursts. Compensating for amplitudes is a critical factor if signals have different frequencies; differences in the amplitudes are more important than differences in the vibration frequencies. If the 235 Hz on one wrist is not compensated (damped accordingly) in the amplitude to match the 185 Hz or 300 Hz signals’ amplitudes, the latter signals can be completely masked.

#### 3.1.2. Ankles

Similar to the wrist position, all subjects could detect the vibration of the 235 Hz continuous signal with the same amplitude on both ankles (session 6). Positioning of transducers is more important than it is for the wrist position: having the motors higher on the lower leg would result in decreased sensitivity. Furthermore, all subjects reported decreased sensitivity on the ankle compared to the wrists in the previous sessions. Signals for sessions 7 and 8 were the same as 4 and 5, but mirrored left and right, and were only completed by 18 subjects. Again, longer bursts were easier to detect, as 12 out of 18 subjects felt a difference, but the result was only 6 out of 18 in the case of the shorter bursts.

#### 3.1.3. Head

In the case of the headband, transducers were placed on the temporal fossa left and right. Unfortunately, these measurement points were insufficient, as none of the subjects could detect vibrations at all (sessions 9–11). Pressing the vibrator to the head with more force resulted in a sensation but also in discomfort. Higher amplitudes and/or different excitation points were suggested for the head. Interestingly, subjects could report when the motor was active, based on auditory evaluation—they could hear the aerial audio signal produced by the vibration (the motors have about a 50 dB sound pressure level at a distance of 0.1 m).

#### 3.1.4. Arms

Placement of the vibrators was on the inner side of the elbow, high up on the lower arm. Only 18 out of 27 subjects reported equal amplitude vibration on both sides using the 235 Hz signal (session 12). The remaining 9 subjects reported different vibration amplitudes on the left and right. All subjects agreed that there was a decreased sensitivity at this point compared to the wrists. The arm is also sensitive to the placement of the transducers. In sessions 13 and 14, longer bursts were easier to detect, as 14 out of 18 subjects felt a difference, but the result was only 7 out of 18 in the case of the shorter bursts.

### 3.2. Four-Channel Experiments

The 7 4-channel sessions were used on the wrists and ankles only. No. 15 was the only mandatory session using 250 ms of 235 Hz bursts (27 answers). Sessions 16 and 17 tested the left–right body symmetry with the same signals. Sessions 18–21 tested whether a shorter burst signal could be detected among three longer burst signals. Similar to the 2-channel sessions, the same distribution of signals was applied to the subjects, thus, these were also completed by 18 subjects. All 27 subjects could identify that it is the same signal at all four excitation points. However, due to the lower sensitivity at the ankles, lower amplitudes were reported here than on the wrists (note: amplitude compensation was only applied using different frequencies in sessions 2 and 3). Furthermore, it was a common subjective comment that this task was more difficult and needed more concentration than the two-channel tests. In the case of No. 16 and 17, a left–right signal presentation was applied, using the same signals on the left ankle/wrist and a different signal on the right ankle/wrist. The 200 ms burst signal could be identified by all 18 subjects (100%) if it was on the left side, but only by 6 persons if it was on the right side. For sessions 18–21, only one channel contained the shorter 200 ms bursts. All 18 subjects were able to answer correctly on the wrists (both left and right side—No. 19 and No. 20). In the case of No. 18 (left ankle) only 12 subjects, and in the case of No. 21 (right ankle) only 6 subjects could answer correctly. Again, decreased performance was observed on the right half of the body.

### 3.3. Eight-Channel Experiments

In total, 20 eight-channel sessions used all excitation points on the body. Only No. 22 was mandatory with 250 ms of 235 Hz bursts (27 answers). All other sessions were distributed among the subjects in a way that we could obtain 9 responses for each session. Sessions 23 and 24 tested left–right symmetry with the same signals. Sessions 25–32 tested whether a longer burst signal could be detected among seven shorter burst signals. Session 33 used P1 on all channels to test the detectability of a simple pattern. Sessions 34–41 tested whether the S.O.S. pattern could be detected among seven other patterns. It is important to note that distractor signals varied over the sessions: no. 34 has the same (P4) distractor on all 7 channels, no. 35 had two different patterns, etc. The number of different distractors increased, and no. 40 and 41 had 7 different sounds on 7 different channels. The main problem with the 8-channel test was that the vibrators fixed to the headband were undetectable as vibration but the signals were audible through the ears. All 27 subjects (session 22) identified the six vibrations on the extremities as identical and reported audible signals at the head. Ankle and arm positions had decreased sensitivity compared to the wrist, these signals appeared to be weaker. Left and right side comparison sessions were performed by 9 subjects. The 250 ms short burst could be identified by all 9 subjects (100%) if it was on the left side, but only by 3 persons if it was on the right side. Sessions 25–32 used seven identical channels of 250 ms burst and only one with a long burst of 500 ms. The task was to identify the channel with the 500 ms bursts. All subjects could identify the long burst signal if it was presented on either of the wrists. This was followed by the ankles: all 9 subjects identified it on the left ankle and 6 out of 9 on the right ankle. Right side results were inferior, similar to the 4-channel situations. Interestingly, the arm positions were unusable, none of the subjects could identify the longer burst signals. Although 2 out of the 9 subjects correctly identified the channel on the headband based on auditory evaluation, this test was labeled as unsuccessful. Sessions 33–43 were the most challenging. First (no. 33), pattern 1 was presented using all channels simultaneously. From the 9 subjects, 6 identified correctly the signals as identical. In sessions no. 34–41, the task was to find the channel with the S.O.S. pattern while being exposed to 7-channel distractor signals. The wrist position seemed to be the most accurate: 4 out of 9 could identify the signal on the right wrist, and 6 out of 9 on the left. In the case of ankles, 5 could identify the signal on the left ankle and 4 on the right ankle. However, the differences in this case were statistically not significant. Arm positions and headband positions were unusable.

## 4. Discussion

**Figure 8:**
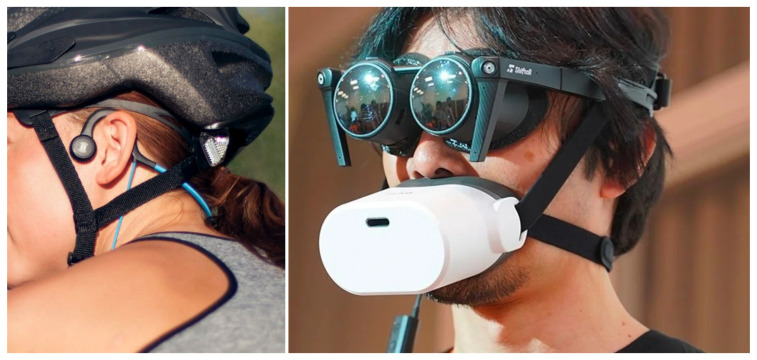
A bone conduction headphone using the bones in the jaw and upper cheek to transmit vibrations directly into the inner ear during playback (**left**) and Mutalk, the latest development for the Metaverse: a microphone with a mute function that prevents one’s voice from leaking out (**right**). Using a helmet instead of glasses will not leave space for further devices to be attached to the head.

All results suggest that the wrist position has the best accuracy in detecting temporal patterns and amplitude levels of the presented signals, followed by the ankles. Moving the transducer position higher up on the lower arm, sensitivity decreases rapidly, and around the elbow (arm position) usability of the signals and/or different patterns becomes insufficient. Ankles are less sensitive than wrists, thus, the same vibrational amplitude causes a weaker sensation. Sensation on the arms versus those on the ankles was not contrasted directly, only in contrast to the wrist measurement points. Informal reports from the subjects indicated almost the same sensitivity, however, ankles were always preferred over arms. The temporal fossa is also an inconvenient placement. Sensation of vibration was impossible due to the weak force that was used to push the transducers to the skull. A greater force could result in signal detection, but was reported as unpleasant and not favored by the subjects due to ergonomic considerations. Although the vibrations could be sometimes detected auditorily, this was regarded as a malfunction and informally labeled as being “rather a bug than a feature”. In a real application (simulators, VR/AR systems), other devices, headsets, headphones, glasses, helmets, etc., are usually also worn to provide visual and auditory information, thus, tactile channels are not preferred on the head. Compared to other areas of the body, tactile research and interfaces for the head are still sparse [10]. Bone conduction devices attached to the jaw bone or the mastoid are commercially available for 2-channel signal reproduction and could be used separately if the skull has to be used for feedback. Figure 8 shows examples of the latest developments in bone conduction playback and for muting human speech during communication in the virtual realm, where one of the major problems is that others can overhear speaking. VR devices will not leave too much space for tactile feedback solutions on the head.

Regarding frequency, two different, amplitude-equalized continuous sinusoidal signals (185 Hz and 300 Hz) were used in only two sessions on the wrists. Only one-third of the subjects could detect any difference in the frequency related to the nominal 235 Hz frequency. This suggests that amplitude is a more important parameter than frequency. In total, 185 Hz and 300 Hz were not compared directly in the experiment. Nevertheless, in an informal side experiment, 10 volunteers tested them on the wrists and 5 could detect “some difference”. Therefore, it is recommended to use only the resonant frequency of a given transducer and to generate alternative excitation signals based on the vibration’s amplitude and temporal patterns. This result supports former findings with 12 subjects focusing on the perception sensitivity of tactile patterns, where participants were able to discriminate 24 tactile patterns with up to 99% accuracy on the wrists and where intensity was more difficult to detect than temporal pattern [62].

Generally, longer bursts of about a minimum of 500 ms were easier to detect than shorter ones. Results of testing of vibrations of different lengths of a mobile device suggested that the optimal duration of vibrations to be around 50–200 ms, and longer durations to be disturbing [28]. Our data did not support this finding.

Subjects reported that they were able to use 2 and 4 channels with ease, which was also supported by the results. Taking accuracy into account, the logical solution for these channels would be the left and right wrist and ankles. Due to good sensitivity on the wrists, any further channels should be attenuated, otherwise it can mask other channels. Masking effects are common in the same modality, but also crossmodal perceptual suppression effects can be demonstrated (e.g., tactile information suppressing visual information) [63,64]. It is recommended to set vibration attenuation individually in order to match it. In the case of the ankles, placement of the band is critical, as it should be right above the medial malleolus of the tibia bone.

In total, 17 out of 27 subjects would prefer a 4-channel presentation, only 4 would prefer the 2-channel presentation, and a remarkably high number of 6 would try it with 6 or 8 channels. This is, however, speculative, as they could not relay anything from the two channels on the head and it was very limited from the arms.

The results support former findings using 8-channel haptic armbands and different vibration patterns, where sensation thresholds depended on actuator positions, and the maximum resolution for stimuli discrimination was 4 [52]. Furthermore, the optimal vibration pattern was different for participants. Although they did not compare different patterns directly in our experiment, subjects were exposed to different patterns during the 8-channel discrimination sessions. Comparison of different patterns and individual preferences is planned for future works. In the case of more than two channels, analyzing and detection are more demanding. It takes more time and cognitive load (it is tiring) and sensory overload could happen.

There is an interesting tendency to have decreased performance on the right side if we have more than 2 channels. A direct comparison was made between the two body sides using the same signals mirrored during sessions 16 and 17 (4-channel) and sessions 23 and 24 (8-channel). We can speculate that an imbalanced performance on the left and right side could be due to left or right-handedness, but we did not ask subjects about this. Furthermore, it was not observed during 2-channel measurements at all. To confirm such assumptions, a larger number of left-handed subjects should be recruited to obtain a statistical significance. To exclude some kind of biased error in the measurement system regarding left- and right-side channels, some sessions in which left/right asymmetries occurred were repeated with reversed channels.

The AC motors of this size performed well. The most important parameter is the nominal voltage, as the largest amplitude can be produced here. In the case of weak amplitudes, additional external signal amplifiers are recommended. Another solution could be to use more transducers for one channel or a larger vibrating surface (note that, in the case of mobile phones, the whole case is vibrating). Vibration thresholds reduce as the contact surface increases, however the decrease is not inversely proportional to the increase in contact area [16].

**Figure 9:**
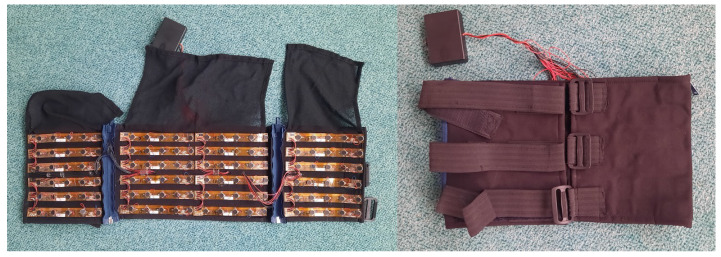
Vest-like feedback device with 4 × 30 vibrators [35,65].

Based on informal communication, 24 subjects would use a system with tactile feedback, where vibrations deliver additional information. The question was not directed to ergonomic aspects, whether long-term use of bands on the wrists and ankles would cause any problems or discomfort. Subjects generally welcomed the idea of having multi-channel tactile feedback options, even those that use more than 4 channels. Furthermore, the current device is wired, which can also limit users’ movements and comfort during usage. Wireless solutions, however, could be tricky, as they require active elements in the receiver, and the number of channels can be limited (i.e., Bluetooth). Alternatively, a vest-like device could be used for one or two channels (left and right side of the body) that have vibrators on the chest. Figure 9 shows a prototype vest from our previous Sound of Vision project. Vests that have a large number of vibrators can be uncomfortable to wear, and sensitivity can be low, especially if clothing or body hair blocks direct contact with the skin.

During multi-channel presentation, especially in the case of 8 channels, subjects reported that their strategy was to first identify the wrist signals, followed by a comparison of the remaining signals in reference to the wrist signals. In the case of left/right symmetries, we can also speculate that subjects figured out the test method, did not use the headband signals at all, and made their 8-channel decisions based on six channels only.

Future research includes an updated measurement setup using different sessions, number of channels, and modified transducer positions (i.e., chest, jaw bone).

## 5. Conclusions

This paper presented experimental results of a multi-channel tactile feedback system designed for applications in assistive technology. In total, 27 volunteers evaluated vibration signals presented on the wrists, ankles, arms, and head using LRA vibrating motors and signals composed of sinusoidal signal bursts and patterns in 41 selected measurement sessions. Results indicate that wrist and ankle measurement points can be used with high accuracy in 2-channel and/or 4-channel presentation, but arms and skull are insufficient and inconvenient excitation points on the body. Signals bursts longer than 500 ms or continuous signals are recommended. Changes in the frequency of the vibration are difficult to detect, thus, variations in the amplitude and/or in temporal properties (patterns) should be used. Further experiments could target relevant research questions about left/right asymmetries, other excitation points on the body, and different vibration patterns.

## Figures and Tables

**Table 1:** Sound card output connectors with the corresponding wiring solutions and transducer placements.

Sound Card Output	Connector Type	Vibrator Position
Front left	RCA	left temporal fossa
Front right	RCA	right temporal fossa
Center	3.5 mm stereo jack (L)	left arm
LFE/sub	3.5 mm stereo jack (R)	right arm
Left surround side	3.5 mm stereo jack (L)	left ankle
Right surround side	3.5 mm stereo jack (R)	right ankle
Left surround back	3.5 mm stereo jack (L)	left wrist
Right surround back	3.5 mm stereo jack (R)	right wrist

## Data Availability

Not applicable.

## Supplementary Materials

The following supporting information can be downloaded at: https://www.mdpi.com/article/10.3390/s22228962/s1, Tables of statistical analysis.

## Abbreviations

The following abbreviations are used in this manuscript:

AT	Assistive Technology
DAW	Digital Audio Workstation
LRA	Linear Resonant Actuator
VPT	Vibration Perception Threshold
