# Supplementary material for: Perception Accuracy of a Multi-Channel Tactile Feedback System for Assistive Technology
Source: Sensors (Basel). 2022 Nov 19;22(22):8962. doi: 10.3390/s22228962 (PMC9695395; doi:10.3390/s22228962)
Supplement: Supplementary file 1 [file sensors-22-08962-s001.zip › sensors-1953795-supplementary.pdf]

Supplementary Material

The following tables summarize results based on statistical analysis for significance. Evaluation was based on nominal (binary) variable analysis using two-sample proportion tests for significance. H_0 is that proportions are equal in the groups.

2-channel

**excitation points**	**task**	**result**	**test statistics**	**significance (p<0.05)**
wrists	detect 235 Hz L and R (S1)	27/27		
	detect 300 Hz on the R (S2)	9/27	p-value: 3.0E-7 z=5.196	YES
	detect 185 Hz on the L (S3)	9/27	p-value: 3.0E-7 z=5.196	YES
	detect shorter on the L (S4)	13/27	p-value: 1.67E-5 z=4.347	YES
	detect longer on the R (S5)	22/27	p-value: 0.02 z=2.348	YES
ankles	detect 235 Hz L and R (S6)	27/27		
	detect longer on the L (S7)	12/18	p-value: 0.012 z=3.222	YES
	detect shorter on the R (S8)	6/18	p-value: 1.0E-6 z=4.954	YES
arms	detect 235 Hz L and R (S12)	18/27	p-value: 0.001 z=3.286	YES
	detect shorter on the L (S13)	7/18	p-value: 0.066 z=-1.837	NO
	detect longer on the R (S14)	14/18	p-value: 0.420 z=0.805	NO

Comparison:
WRIST
Detecting 185 Hz and 300 Hz compared to detecting 235 Hz: significant (p-value: 3.0E-7, z=5.196152)
Detecting shorter on the left compared to detecting the normal: significant (p-value: 1.67E-5, z=4.347413)
Detecting longer on the right compared to detecting the normal: significant (p-value: 0.02, z=2.34738)
Detecting shorter on the left compared to detecting longer on the right: significant (p-value: 0.018, z=2.3473)

ANKLES
Detecting longer on the left compared to detecting the normal: significant (p-value: 0.012 z=3.222)
Detecting shorter on the right compared to detecting the normal: significant (p-value: 1.0E-6, z=4.954)
Detecting shorter on the right compared to detecting longer on the left: significant (p-value: 0.045, z=2.000)

ARMS
Detecting 235 Hz on the arms compared to on the wrists and ankles: significant (p-value: 0.001, z=3.286)
Detecting shorter on the left compared to detecting the normal: not significant (p-value: 0.066, z=-1.837)
Detecting longer on the right compared to detecting the normal: not significant (p-value: 0.420, z=0.805)
Detecting shorter on the left compared to detecting longer on the right: significant (p-value: 0.018, z=-2.366)

4-channel

**excitation points**	**task**	**result**	**test statistics**	**significance ($p<0.05$)**
wrists and ankles	detect 235 Hz (S15)	27/27		
wrists and ankles	detect shorter on the R (S16)	6/18	p-value: 1.0E-6 z=4.954	YES
wrists and ankles	detect shorter on the L (S17)	18/182		
left ankle	detect shorter (S18)	12/18	p-value: 0.007 z=2.683	YES
left wrist	detect shorter (S19)	18/18		
right wrist	detect shorter (S20)	18/18		
right ankle	detect shorter (S21)	6/18	p-value: 1.0E-6 z=4.954	YES

Comparison:
Detecting shorter on the left compared to detecting the normal: not significant (p-value: 1)
Detecting longer on the right compared to detecting the normal: significant (p-value: 1.0E-6, z=4.954)
Detecting shorter on the left compared to detecting longer on the right: significant (p-value: 2.89E-5, z=4.242)
Detecting shorter signals on the ankles compared to normal signals on the wrists (left and right): significant (p-value: (1.0E-6, z=4.954); (p-value: 0.007, z=2.683)

8-channel

**excitation points**	**task**	**result**	**test statistics**	**significance (p<0.05)**
all channels	detect 235 Hz (S22)	27/27		
all channels	detect shorter on the R (S23)	3/9	p-value: 3.0E-6 z=4.648	YES
	detect shorter on the L (S24)	9/9		
left ankle	detect longer (S27)	9/9		
left wrist	detect longer (S28)	9/9		
right wrist	detect longer (S29)	9/9		
right ankle	detect longer (S30)	6/9		
arms and head	detect longer (S25, S26, S31, S32)	0		
all channels	detect P1 pattern (S33)	6/9	p-value: 0.002 z=-3.133	YES
left ankle	detect S.O.S. (S36)	5/9		NO
left wrist	detect S.O.S. (S37)	6/9		NO
right wrist	detect S.O.S. (S38)	4/9		NO
right ankle	detect S.O.S. (S39)	4/9		NO
arms and head	detect S.O.S. (S34, S35, S40, S41)	0		

Comparison:
Detecting shorter on the left compared to detecting the normal: not significant (p-value: 1**)**
Detecting longer on the right compared to detecting the normal: significant (p-value: 3.0E-6, z=4.648)
Detecting shorter on the left compared to detecting longer on the right: significant (p-value: 0.003, z=3.000)
Detecting 235 Hz compared to P1: significant (p-value: 0.002, z=-3.133)
